# Effects of the Primary NbC Elimination on the SSCC Resistance of a HSLA Steel for Oil Country Tubular Goods

**DOI:** 10.3390/ma14185301

**Published:** 2021-09-14

**Authors:** Tianyi Zeng, Shuzhan Zhang, Xianbo Shi, Wei Wang, Wei Yan, Yan Tian, Mingchun Zhao, Ke Yang

**Affiliations:** 1 Shi-Changxu Innovation Center for Advanced Materials, Institute of Metal Research, Chinese Academy of Sciences, Shenyang 110016, China; tyzeng19b@imr.ac.cn (T.Z.); szzhang20b@imr.ac.cn (S.Z.); wangw@imr.ac.cn (W.W.); weiyan@imr.ac.cn (W.Y.); kyang@imr.ac.cn (K.Y.); 2 School of Materials Science and Engineering, University of Science and Technology of China, Hefei 230026, China; 3 CAS Key Laboratory of Nuclear Materials and Safety Assessment, Institute of Metal Research, Chinese Academy of Sciences, Shenyang 110016, China; 4 School of Materials Science and Engineering, Central South University, Changsha 410083, China; tianyan0905@163.com (Y.T.); mczhao@csu.edu.cn (M.Z.); 5 Hengyang Valin Steel Tube Co., Ltd., Hengyang 421001, China

**Keywords:** SSCC, HSLA steels, electroslag remelting, continuous casting, primary NbC carbides

## Abstract

Sulfide stress corrosion cracking (SSCC) has been of particular concern in high strength low alloyed (HSLA) steels used in the oil industry, and the non-metallic inclusions are usually considered as a detrimental factor to the SSCC resistance. In the present work, continuous casting (CC) and electroslag remelting (ESR) were adopted to fabricate a 125 ksi grade steel in order to evaluate the effect of microstructure with and without primary NbC carbides (inclusions) on the SSCC resistance in the steel. It was found that ESR could remove the primary NbC carbides, and hence, slightly increase the strength without deteriorating the SSCC resistance. The elimination of primary NbC carbides caused two opposite effects on the SSCC resistance in the studied steel. On the one hand, the elimination of primary NbC carbides increased the dislocation density and the proportion of high angle boundaries (HABs), which was not good to the SSCC resistance. On the other hand, the elimination of primary NbC carbides also induced more uniform nanosized secondary NbC carbides formed during tempering, providing many irreversible hydrogen traps. These two opposite effects on SSCC resistance due to the elimination of primary NbC carbides were assumed to be offset, and thus, the SSCC resistance was not greatly improved using ESR.

## 1. Introduction

With the increasing exploitation of shallow oil and natural gas, some deep wells and ultra-deep wells have recently been developed in some countries [1]. In these wells, both a high pressure and a high temperature caused detrimental effects on the drilling and production equipment. More seriously, there was wet corrosive gas such as hydrogen sulfide (H_2_S) contained in fossil fuels, and serious corrosion problems and resultant sulfide stress corrosion cracking (SSCC) might generate in a sour environment where H_2_S exists [2]. As a form of hydrogen embrittlement (HE), the occurrence mechanism of SSCC has been illustrated for years [3,4,5,6]. Basically, SSCC phenomenon will take place when the high strength steels suffer from the joint action of external tensile stress and a specific corrosive environment where the solution pH value is low and the H_2_S partial pressure is high. The whole process usually involves several stages in sequence [7]. Firstly, the corrosion of steel occurs in a sour environment with the formation of iron sulfide scales on the surface of steel. Secondly, pitting corrosion takes place due to the galvanic coupling steel-scale, followed by H atoms permeation into steel. After that, cracks initiate at the bottom of the pits and then propagate with the help of H atoms and external stress. At last, fracture breaks out without warning.

Undoubtedly, SSCC will result in enormous economic loss and serious environmental pollution, which should be prevented in advance. It was widely accepted that the SSCC resistance of steels greatly depends on the amount of freely diffusing H and its effective diffusivity, both of which are related to the microstructure [8]. Moreover, the contradiction between strength and SSCC resistance was always hard to coordinate in HSLA steels [9,10]. To increase the strength without deteriorating SSCC resistance, Nb alloying has been widely used in HSLA steels for the refinement of grains and the formation of nanosized NbC carbides, both of which are considered to alleviate the susceptibility of SSCC by introducing strong irreversible H traps and impeding the H enhanced localized plasticity [11,12,13]. However, both Nb and C are easily-segregated elements, and the primary NbC carbides are likely to form during casting [14,15], and this type of coarse incoherent NbC was thought impossible to trap hydrogen atoms effectively [16]. Besides, it was reported that non-metallic inclusions, as the potential cracking sources, play a significant role in decreasing brittle fracture resistance of steels [17]. Consequently, the removement of primary NbC carbides becomes important in terms of increasing coherent NbC carbides as strong H traps during tempering or improving brittle fracture resistance of steels. Moreover, the elimination of primary NbC carbides also enables more C and Nb to be dissolved in austenite, and the consequent changes in the microstructure related to SSCC resistance after quenching and tempering need to be examined. Compared with CC, the electroslag remelting (ESR) technology in steelmaking industry is generally characterized as a very fast cooling rate after casting [18], and thus the segregation of alloying elements and carbon can be decreased to a certain extent. Consequently, in this work, CC and ESR were used to study the effect of microstructure with and without primary NbC carbides on the SSCC resistance in a 125 ksi grade steel.

## 2. Materials and Methods

A 125 ksi grade HSLA steel was prepared by conventional continuous casting and electroslag remelting (henceforth referred to as Steel CC and ESR, respectively). The metallurgical process of Steel CC was composed of electric arc furnace (EAF) melting → ladle furnace (LF) refining → vacuum degassing (VD) → CC, and the metallurgical process of Steel ESR was composed of EAF → LF → VD → CC → ESR. After casting, the ingots with 280 mm diameter for each steel were immediately kept at 1240 °C for 3 h in a rotary furnace, followed by hot-piercing process, rolling process and stretch-reducing rolling process, as described in the previous study [19]. The finish rolling temperature was about 810 °C, at which the wall thickness of the tubes was reduced to about 29.5 mm. The chemical compositions of tubes from Steel CC and ESR were shown in Table 1. Subsequently, the samples from tubes for each steel were quenched into water after austenitizing at 910 °C for 1 h, and then a high temperature tempering treatment was immediately performed at 690 °C for 1 h, followed by water cooling. The microstructural characterizations were performed on the cross-section of tubes. The primary NbC carbides were examined using a FEI INSPECT field-emission scanning electron microscope (FE-SEM, TESCAN MIRA3, Brno, Czech) on samples etched in 4% nital, and their semi-quantitative element distribution was examined by energy dispersive X-ray spectroscopy (EDS, X-Max^N^, Oxford Instruments, UK) attached to the FE-SEM. For measurements of prior austenite grain size (PAGS), the as-quenched samples were initially heat-etched in the saturated picric acid aqueous solution, and, then, the PAGS was measured on an optical microscope (OM, LEICA DMIRM, Jena, Germany) using a linear intercept procedure. The as-tempered microstructure was characterized using OM and SEM after etching in 4% nital. Electron backscatter diffraction (EBSD, NordlysMax^3^, Oxford Instruments, UK) measurements were performed on two randomly selected regions in the as-tempered samples which were electropolished with an electrolyte (90% methanol and 10% perchloric acids in volume) at 30 V for about 5 min at −40 °C. Secondary NbC carbides formed during tempering was characterized on the thin foils through a TECNAI F20 transmission electron microscope (TEM, FEI, Oregon, USA). The average diameter of Cr-rich carbides was measured using a commercial Image-Pro Plus software (version 6.0, Media Cybernetics, Georgia, USA) based on five randomly selected SEM images.

X-ray diffraction (XRD) line profiles were obtained from the as-tempered samples and annealed Si powders using a Rigaku D/max 2500 PC diffractometer (Ni-filtered Cu *Kα* radiation, λ = 0.15405 nm, Rigaku Corporation, Tokyo, Japan) with an angular range of 40°~140° (2θ) and a step interval of 0.01° (2θ). The dislocation densities in Steel CC and ESR were evaluated by the convolutional multiple whole profile (CMWP) fitting procedure. In CMWP, the peak profile functions were firstly assumed to be the convolution of the grain size, strain, and instrumental broadening (evaluated using annealed Si powders), and then the measured XRD profile was fitted with the calculated peak profile functions based on a non-linear least-squares method [20,21,22]. The volume fraction of retained austenite was calculated according to the ASTM E975-03 standard [23].

The tensile properties were examined at room temperature using three samples with 5 mm gauge diameter and 60 mm gauge length, and the impact toughness was tested at 0 °C using three samples with 10 mm square in cross-direction, 2 mm in V-notch depth, and 55 mm in length. The specimens for tensile tests were cut from the longitudinal direction of the tubes. The resistance to SSCC was evaluated using uniaxial constant load test according to NACE TM 0177-2005 Method A, and the specimens with 6.35 mm gauge diameter and 25.4 mm gauge length were extracted from the longitudinal direction of the tubes. With bearing a uniaxial tensile stress of 85% and 80% specified minimum yield strength (SMYS), respectively, the specimens were immersed in the solution A with 0.1 MPa H_2_S saturated at room temperature. The SMYS herein was arbitrarily determined to be 758 MPa. The solution A consisted of an acidified aqueous solution containing 50 g/L NaCl and 5 g/L of CH_3_COOH with an initial pH value of around 2.7. The recorded failure time of samples was used to evaluate the SSCC resistance for each steel. Moreover, a commercial Thermo-Calc software (version 3.0.1, Thermo-Calc Software, Stockholm, Sweden) with TCFE 7 database was used to simulate the solidification process of the studied steel, and the volume fractions of various phases at a temperature were also calculated assuming that the thermodynamic equilibrium was achieved.

## 3. Results

### 3.1. Simulated Solidification Process Using Thermo-Calc

**Figure 1:**
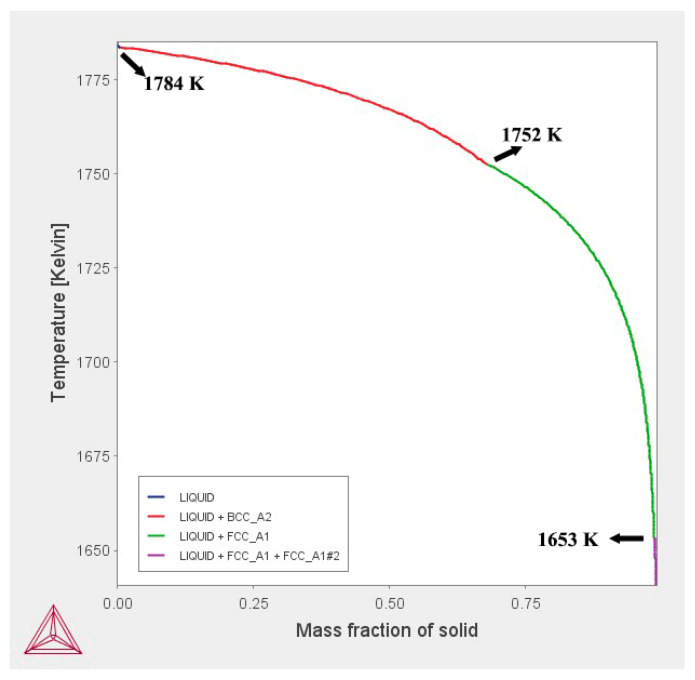
Simulative solidification process of the Steel CC.

Based on the Scheil module integrated in Thermo-Calc software using TCFE 7 database, the formation of primary NbC carbides during casting could be simulated as shown in Figure 1. It can be found that the studied steel melt starts to solidify at about 1784 K, and the content of BCC_A2 (δ) phase gradually increases as the temperature decreases. When the temperature decreases down to 1752 K, the FCC_A1 (γ) phase will form. Subsequently, the FCC_A1#2 (NbC) phase forms at 1653 K. The content of FCC_A1#2 (NbC) phase increases as the solidification process proceeds until the liquid phase is fully consumed. The mass fraction of primary NbC carbides (FCC_A1#2) is determined to be 0.01 mass%.

### 3.2. Microstructural Characterization

#### 3.2.1. Primary NbC Carbides and Prior Austenite Grain Size

**Figure 2:**
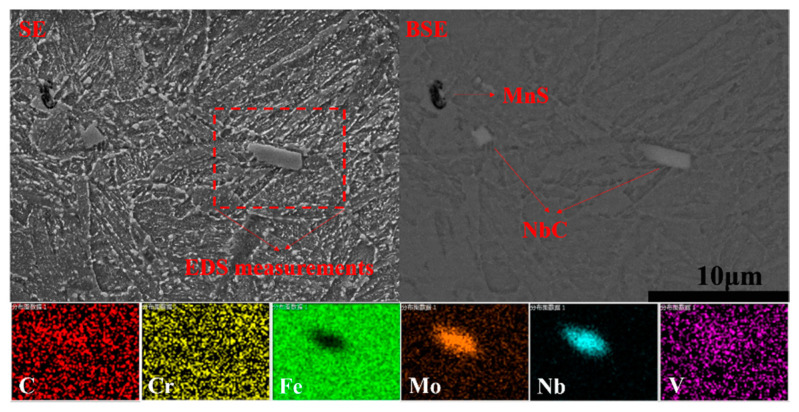
SEM images showing the primary NbC carbides in Steel CC.

Notwithstanding the similar C and Nb contents, the precipitation behavior of the primary NbC carbides varied greatly in the steels CC and ESR. The micronsized granular primary NbC carbide was not found in Steel ESR but in Steel CC, as shown in Figure 2. By using SEM, the backscattered electron (BSE) image could clearly exhibit primary NbC carbides in contrast to the secondary electron (SE) image due to its significant contrast, and the EDS measurements proved that Mo segregated into the primary NbC carbides as well. Moreover, MnS inclusions usually located in the segregation areas in Steel CC, while it was hard to find MnS inclusions in the steel prepared by ESR, which is due to desulfurization in ESR. As shown in Table 1, the weight percent of S was only 0.0007 wt.% in Steel ESR, which was lower than that in Steel CC by an order of magnitude, showing a strong desulfurization ability of ESR.

**Figure 3:**
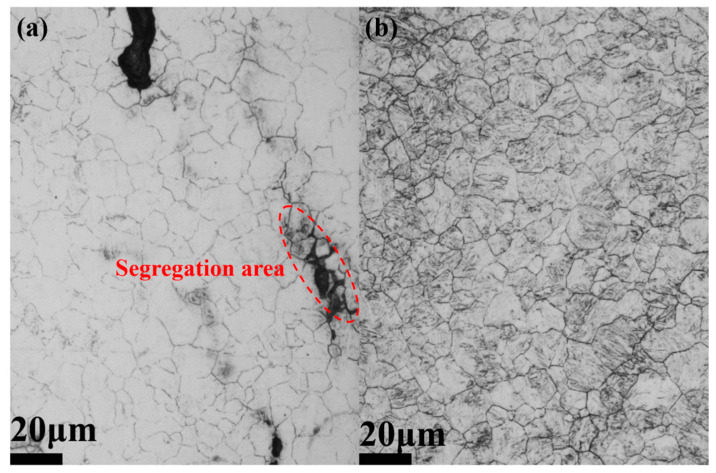
OM images showing the PAGS in the as-quenched steels (**a**) CC and (**b**) ESR.

As shown in Figure 3a, a refinement effect was found on the grains in the segregation areas due to the precipitation of primary NbC carbides. However, as Figure 3b shows, more uniform prior austenite grains formed in Steel ESR, which was possible due to the decrease of Nb segregation. The average PAGSs of two steels were determined to be 10.1 ± 0.3 μm and 9.9 ± 0.2 μm for Steel CC and Steel ESR, respectively, which implies that there was no great difference in PAGS between these two steels.

#### 3.2.2. Various Carbides Formed during Tempering

**Figure 4:**
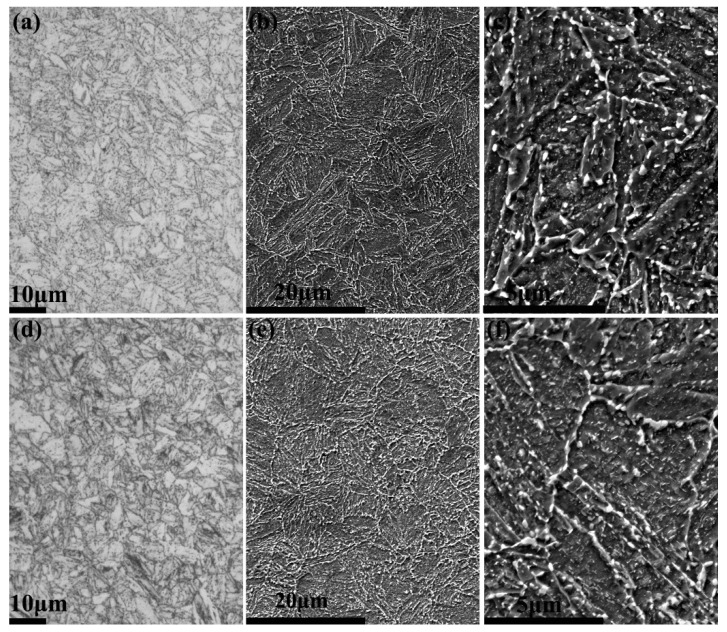
OM images (**a**,**d**) and SEM images (**b**,**c**,**e**,**f**) showing microstructures in the as-tempered steels CC (**a**–**c**) and ESR (**d**–**f**).

**Figure 5:**
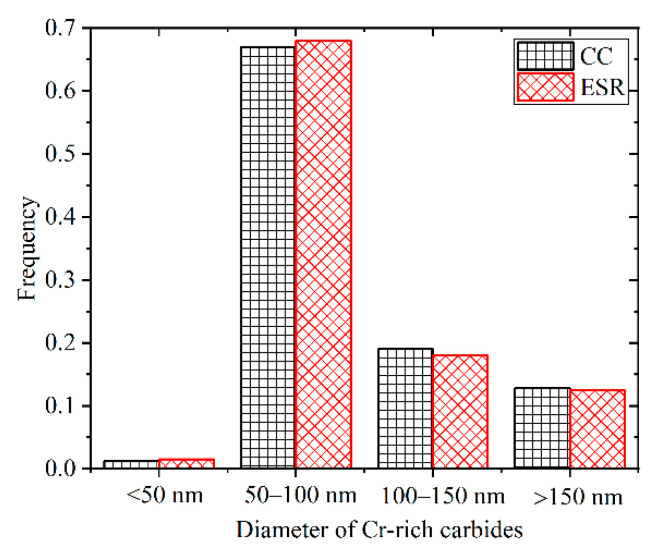
Size distribution frequencies of Cr-rich carbides in the as-tempered steels CC and ESR.

The microstructures of two steels after tempering at 690 °C were lath martensite, as shown in Figure 4. It was shown that some nanosized carbides formed at lath, block, packet and prior austenite grain boundaries, and a large number of finer carbides tended to precipitate within laths. Based on the subsequent TEM characterization, these carbides were mainly characterized as Cr-rich carbides. By randomly selecting five SEM images, the sizes of carbides in Steels CC and ESR were measured using the Image-Pro Plus 6.0 software, and the sizes distribution of carbides was described in Figure 5. The carbides size was arbitrarily divided into four categories, and no apparent discrepancy in the size distribution of carbides was found between Steels CC and ESR. Moreover, the average carbide sizes were calculated to be 98 ± 10 nm and 95 ± 8 nm for Steel CC and ESR, respectively.

**Figure 6:**
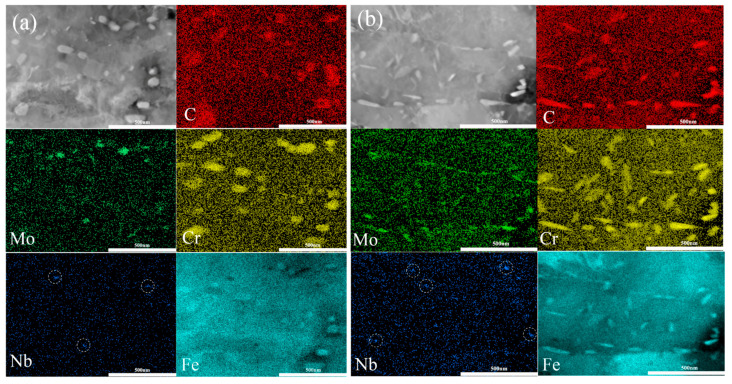
TEM images showing various carbides in the as-tempered steels (**a**) CC and (**b**) ESR.

To examine the, probable, much finer carbides such as VC and secondary NbC carbides, TEM characterizations were performed. As shown in Figure 6, there were various carbides in the steels CC and ESR. More specifically, those comparatively large carbides were mainly Cr-rich carbides, and it was found that the number of nanosized NbC with diameter 20~30 nm was higher in steel ESR than steel CC, as marked by white circles. Moreover, these small NbC carbides were much smaller than those primary counterparts found in the segregation areas.

#### 3.2.3. EBSD Characterization

The crystallographic features have been known to play an important role in the interaction of hydrogen with steels [6]. As an effective technique, EBSD is usually used to detect different boundaries and local strain distributions in the as-tempered martensite by measuring the misorientation angle between adjacent points in a grain [24]. In general, the grain boundaries are classified into two groups: low angle boundaries (LABs, 2° ≤ θ < 15°) and high angle boundaries (HABs, θ ≥ 15°). Moreover, the HABs having coincidence site lattice (CSL) are also defined as “special” grain boundaries with few crystalline defects [25]. In this work, the grain boundary character distribution (GBCD) was calculated in terms of LABs, HABs, and CSLs.

**Figure 7:**
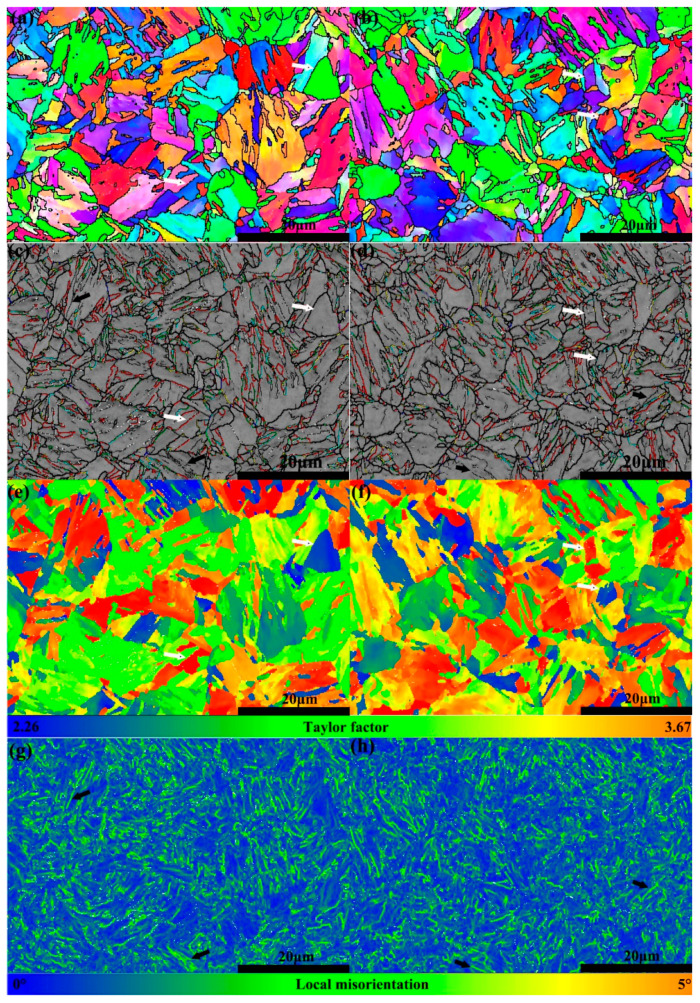
EBSD results of as-tempered Steels (**a**,**c**,**e**,**g**) CC and (**b**,**d**,**f**,**h**) ESR, in which (**a**,**b**) are orientation maps, (**c**,**d**) are band contrast maps decorated with grain boundaries, (**e**,**f**) are Taylor factor maps, and (**g**,**h**) are local average misorientation maps (in (**a**,**b**), the black line is HABs; in (**c**,**d**), the fine black line and thick black line are LABs and HABs, respectively, and the colored lines are CSLs).

As shown in Figure 7a,b, a prior austenite grain was divided into several packets according to different habit planes, and a packet is further subdivided into some blocks according to different habit orientations, as well described by the work of Morito and co-workers [26]. Different grain boundaries are shown in Figure 7c,d, and some white dots which mainly situated on boundaries were zero resolution. It shows that the colored lines, which represent different CSLs with various sigma values, overlap with the HABs. As shown in Figure 7e,f, a prior austenite grain was possibly decorated by various colors according to different Taylor factor values. The average Taylor factors were 3.01 ± 0.13 and 3.07 ± 0.15 for Steel CC and ESR, respectively. The color-coded local misorientation maps of the studied steels were demonstrated in Figure 7g,h, which can be used to evaluate the local strain distribution.

**Figure 8:**
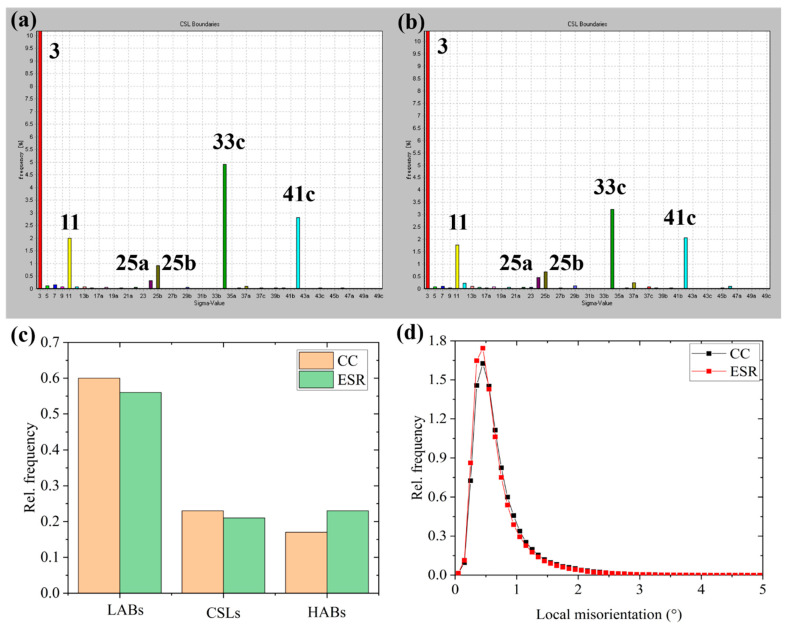
Coincidence site lattice (CSL) boundaries histograms of the steels (**a**) CC and (**b**) ESR, (**c**) relative frequency of GBCD, and (**d**) relative frequency vs. local misorientation of the studied steels.

As shown in Figure 8a,b, the CSL grain boundaries mainly consist of ∑3, ∑11, ∑25a, ∑25b, ∑33c, and ∑41c, and several types CSL boundaries such as ∑3, ∑11, ∑25a, and ∑25b have a similar frequency in Steel CC and ESR, while the frequency of ∑33c and ∑41c are slightly higher in Steel CC than ESR. The statistical relative frequency of the GBCD in two steels is exhibited in Figure 8c. The CSLs and LABs had a higher frequency in Steel CC than Steel ESR, while the HABs displayed a higher frequency in Steel ESR than Steel CC. As for local misorientation, the two curves showing the change of relative frequency with local misorientation are shown in Figure 8d. The peak at 0.5° is higher in Steel ESR than CC, suggesting a higher dislocation density in Steel ESR than CC.

#### 3.2.4. XRD Characterization

**Figure 9:**
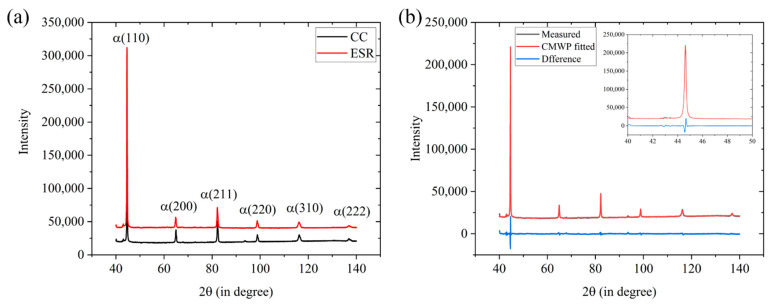
(**a**) XRD profiles measured in the studied steels and (**b**) CMWP fitted XRD profile of Steel CC.

As a product due to incomplete martensite transformation, austenite is usually preserved depending on the martensite-start temperature (M_s_ point). Given that such low carbon content in the current steels, their M_s_ point should be much higher than the room temperature according to some empirical formulas for calculating the M_s_ point [27,28], in which martensite transformation should be finished theoretically. However, due to the C enrichment in surrounding austenite around the formed martensite, there is always the film-type retained austenite in the as-quenched low carbon steels [29]. Besides, reverted austenite probably forms during high temperature tempering, resulting from the local segregation of austenite forming elements that decrease the austenite transformation temperature, which is often the case in martensitic stainless steels [30,31]. The existence of austenite in the as-tempered martensite not only affects the strength and toughness, but also causes a strong effect on diffusivity and solubility of hydrogen [32]. As Figure 9a shows, XRD profiles of two steels after tempering were used to assess the volume fraction of retained austenite. The calculated austenite contents were 1.9 vol.% and 2.3 vol.% for steels CC and ESR, respectively. Because both values were situated in the detection limit on the XRD (1~5 vol.%), it was considered that no austenite formed in two as-tempered steels. Moreover, the XRD profiles of two steels were fitted using the CMWP program, and the fitted curve and measured XRD profile of Steel CC were compared in the Figure 9b, showing a small difference. The calculated dislocation densities were 9.3 × 10^14^/m^2^ and 12.0 × 10^14^/m^2^ for Steel CC and ESR, respectively.

### 3.3. Mechanical Properties and SCC Resistance

The mechanical properties of two steels are shown in Table 2. Both yield strength (YS) and tensile strength (TS) were slightly higher in Steel ESR than Steel CC, while the elongation (A) and Charpy impact energy (KV_2_) were mildly higher in Steel CC than Steel ESR. As for SSCC resistance, the failure time was consistent for two steels with the same applied stress, showing a similar susceptibility to SSCC for two steels.

## 4. Discussion

According to the density of the steel (7.87 g/cm^3^) and NbC (7.82 g/cm^3^) [33], the volume fraction of primary NbC carbides could be calculated to be 0.01% in the studied steels according to its mass fraction. Moreover, the volume fraction of NbC at 690 °C could be calculated to be 0.04% using Thermo-Calc software if the thermodynamic equilibrium state was assumed to be reached. That means the primary NbC carbides accounted for about a quarter of total NbC phase. Considering nanosized NbC carbides formation during tempering, it can be speculated that a quarter of total NbC carbides changing from coarse primary NbC carbides to nanosized NbC carbides in a dispersed distribution. Moreover, considering the chemical formula of NbC and the mass fraction of primary NbC (0.01 mass%), the Nb content in the primary NbC carbides was calculated to be 0.008 wt.%. In other words, there was an extra dissolved 0.008 wt.% Nb in the steel matrix after casting.

**Figure 10:**
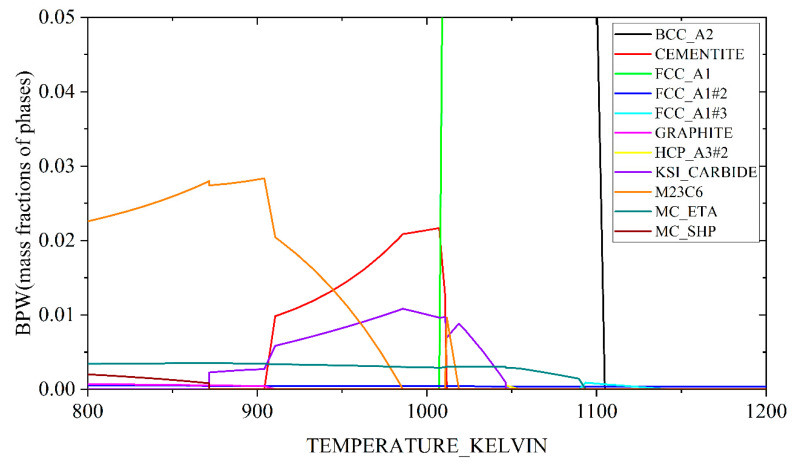
Phase diagram of Steel CC in the temperature range of 800~1200 K.

Based on the thermodynamic equilibrium calculation using the Thermo-Calc software, the evolution in mass fractions of phases (BPW, defined by Thermo-Calc) for Steel CC during 800~1200 K is shown in Figure 10. At the current austenitizing temperature (1183 K), a small quantity of NbC carbides (FCC_A1#2) still existed in the austenite (FCC_A1), due to its high thermodynamic stability. These NbC carbides (FCC_A1#2) usually exerts a positive effect on the refinement of grains by a pinning effect during austenitizing. As previously described, the existence of primary NbC carbides enabled grains in the segregation areas to be finer. However, no significant difference was found in PAGS between the two steels. Two possible reasons could account for this phenomenon. Firstly, Wu and co-workers claimed that Nb atoms in solid solution tended to segregate at grain boundaries and hence, retarded the prior austenite grain growth due to the solute drag effect [34]. In the Steel ESR, there were more Nb atoms in solid solution in spite of the lack of primary NbC carbides, theoretically producing a stronger solute drag effect. Secondly, the ESR generally produces a finer as-cast microstructure than the CC due to a rapider cooling rate [18]. Although NbC carbides seemed to produce a stronger pinning effect on the growth of prior austenite grains in the segregation area, a comparatively weak solute drag effect by Nb was provided in those non-segregation areas. As a consequence, a similar PAGS in steels CC and ESR could be attributed to a joint effect of primary NbC carbides, Nb atoms in solid solution and a finer as-cast microstructure after rapid cooling.

According to Figure 10, when the studied steels were tempered at 690 °C (i.e., 963 K), there were several precipitation phases in the BCC matrix, which were mainly cementite, M_23_C_6_, MC, and so forth. However, based on the EDS results and our previous work [22], the Cr-rich carbides should dominate. It was reported that strong carbide forming elements such as Nb could effectively refine the size of Cr-rich carbides by preferentially combining with C in solid solution to form carbides [35]. However, there was no difference in the size of Cr-rich carbides for two steels despite extra 0.008 wt.% Nb dissolved in Steel ESR. It was speculated that no matter the NbC carbides formed during casting or tempering, the C consumptions for NbC carbides formation were similar in both steels, thus inducing a similar mean size of Cr-rich carbides.

As for EBSD results, it was found that the average Taylor factors in two steels were consistent. As Taylor suggested [36], the Taylor factor is a geometric factor that evaluates the trend of a crystal to slip concerning the orientation of the crystal relative to the sample reference system. Grains with low values of Taylor factor are considered to have “soft” orientations with regard to the given loading direction, favoring the slip activation. Conversely, those grains with high values of Taylor factor are considered to have “hard” orientations with regard to the given loading direction, which is less likely to yield [37]. As shown in Figure 7e,f, a grain colored with blue (Taylor factor with low value) and red (Taylor factor with high value) was marked by white arrows, respectively, and it was found that the grain colored either by blue or by red corresponded to the packet in Figure 7a,b. As Ungar et al. claimed that [38], packets in lath martensite oriented unfavorably or favorably for dislocation glide turn into hard or soft. Consequently, the packet can be regarded as the effective unit when evaluating the deformation capability. For local misorientation distribution, Zhang et al. [11] suggested that the lath/block/packet boundaries showed higher local misorientation values than the matrix, while Liu et al. [24] found high local misorientation values mainly located in the LABs or in the vicinity of LABs. In the current work, as marked by black arrows in Figure 7c,d,g,f, the high accumulation area with a high local misorientation was mainly situated in the LABs, indicating a higher local strain distribution in LABs. Moreover, in regards to CSLs, Zhang et al. [11] argued that a 0.02 wt.% Nb addition could greatly reduce the frequency of ∑3, but there was no great difference in the frequency of ∑3 between two steels, indicating that extra 0.008 wt.% Nb dissolved in Steel ESR had no effect on the frequency of ∑3.

For XRD characterization, due to the elimination of primary NbC carbides, there were more Nb and C contents which strengthened austenite. When the martensite nucleated and propagated into the rest austenite during quenching, strong dislocation multiplication took place in the strengthened austenite, and these dislocations were inherited by subsequently formed martensite, thus introducing more dislocations in Steel ESR than Steel CC.

Due to the similar PAGS and Cr-rich carbides size and the small amount of NbC carbides, the strengthening effect by precipitates and grain boundaries were deemed to be similar in two steels. Moreover, high temperature tempering weakened the solid solution strengthening by C due to the formation of carbides. Besides, considering the similar alloying elements in the steels, the difference in strength was mainly attributed to the dislocation strengthening. The dislocation strengthening (σ_dis_) can be calculated using the following equation [39]:

*σ*_dis_ = *MαGbρ*^1/2^ (1)

where *M* is the Taylor factor (three for bcc polycrystalline materials [40]), *α* is a constant, *G* is the shear modulus of the matrix (76 GPa [41]), *b* is the magnitude of the Burgers vector (0.25 nm [39]), and *ρ* is the dislocation density. As the reference [40] suggested, 0.24 was used as the *α* value in Equation (1). The calculated *σ*_dis_ for the steels CC and ESR were 417 MPa and 474 MPa, respectively, and a difference in calculated *σ*_dis_ (57 MPa) was higher than the difference in the measured yield stress of two steels with only 20 MPa. It was usually accepted that the dislocation strengthening is the largest contribution to the yield strength in lath martensite. Chen et al. found that the dislocation strengthening accounts for about 60% of total yield strength based on the calculation of various strengthening mechanisms [42]. Additionally, Shamsujjoha claimed that dislocation strengthening, as the most important factor, contributed up to 65% of the yield strength [43]. In the current work, the calculated *σ*_dis_ accounted for 48% and 52% of total yield strength in the cases of the steels CC and ESR, respectively, which is close to the reported percentage in references [42,43]. Consequently, the increase in yield strength in Steel ESR was mainly from the dislocation strengthening.

In terms of the SSCC resistance in steels, several factors, such as strength, chemical composition, microstructure, GBCD, Taylor factor (texture), precipitates, and inclusions, were reported to have an influence on H atoms interacting with steel matrix [6]. First of all, the two steels prepared by CC and ESR had the same chemical composition except the S content. A very small amount of MnS inclusions were found in Steel CC, whereas MnS inclusions were absent in Steel ESR, as shown in Figure 2. It was reported in the work [44] that MnS inclusions are preferential H trapping sites, but this conclusion was drawn based on relatively high S content. Given that extreme low S contents in both studied steels, the effect of MnS inclusions on SSCC resistance is thought to be small. Secondly, due to very small austenite contents, the same as-tempered matrix and the similar Taylor factor, the effects caused by these factors on SSCC resistance were considered to be small. For GBCD, low energy LABs are less sensitive to hydrogen attack and crack propagation [25], and LABs and CSLs stop H atoms from building up to a critical level in which H assisted cracks to initiate. In the contrary, high stored energy HABs, acting as H trapping sites, can absorb more H than LABs, providing an easier path for crack growth [13]. Based on the data showed in Figure 8c, the total frequency of LABs and CSLs were higher in Steel CC (0.83) than ESR (0.77), and the frequency of HABs was lower in Steel CC (0.17) than ESR (0.23), inferring a detrimental effect caused by ESR on SSCC resistance in regards to GBCD. Moreover, there was a similar size distribution of Cr-rich carbides in two steels, but more nanosized NbC carbides precipitated during tempering are shown in Figure 6. M. Ohnuma et al. [45] confirmed hydrogen trapped by nanosized NbC in steel using small-angle neutron scattering, and the irreversible hydrogen tapping in nanosized NbC/BCC iron semi-coherent interfaces was proved in the work of Shi et al. [46]. Consequently, the dispersed nanosized NbC carbides formed during tempering contributed a beneficial effect on SSCC resistance. In addition, dislocations in steels were usually present as reversible H traps [13]. More importantly, the dislocations might transport H atoms to those weak areas (such as prior austenite grain boundaries and micro-voids) which were susceptible to SSCC [47]. The more dislocations existed in the steel, the higher SSCC susceptibility would be induced. Consequently, a higher dislocation density induced by ESR not only increased the yield strength by about 40 MPa but also triggered a higher sensitivity to SSCC. Take all factors into account, ESR produced a higher dislocation density, yield strength and a higher frequency of HABs which decreased the SSCC resistance, and induced more nanosized NbC carbides formed during tempering which was good to the SSCC resistance. However, as a whole, the elimination of primary NbC carbides by ESR did not improve the SSCC resistance greatly compared with CC.

## 5. Conclusions

In this work, a 125 ksi grade HSLA steel was prepared by CC and ESR, respectively. The microstructures, mechanical properties and SSCC resistance were studied. Some conclusions could be drawn as follows:

1. The primary NbC carbides were removed by ESR, and hence the yield strength slightly increased without deteriorating the SSCC resistance.
2. The elimination of primary NbC carbides increased the dislocation density and the proportion of high angle boundaries (HABs), which was not good to the SSCC resistance.
3. The elimination of primary NbC carbides promoted a more uniform nanosized secondary NbC carbides formation during tempering, providing many irreversible hydrogen traps.
4. The opposite effects on SSCC resistance resulted from the increase of dislocation density and the proportion of HABs as well as the formation of more nanosized secondary NbC carbides were assumed to be offset, and thus the SSCC resistance was not greatly improved using ESR.
5. In view to the experimental results and the practical cost of performing ESR, this work, as a guidance for industrial application, suggested that it was not necessary to use ESR to produce HSLA steels for OCTGs as well as pipelines.

## Figures and Tables

**Table 1:** Chemical compositions of the studied steels (in wt.%).

Steels	C	Nb	V	Mo	Mn	Cr	Si	Al	Ti	N	P	S
CC	0.26	0.029	0.08	0.78	0.43	0.55	0.22	0.031	0.003	0.007	0.010	0.0023
ESR	0.25	0.026	0.08	0.78	0.42	0.54	0.25	0.036	0.004	0.007	0.009	0.0007

**Table 2:** Mechanical properties and SSCC resistance of the studied steels.

Steels	Mechanical Properties				SSCC Resistance	
	YS (MPa)	TS (MPa)	A (%)	KV_2_ (J)	% SMYS and Applied Stress	Failure Time (h)
CC	868 ± 9	918 ± 10	19.5 ± 1.4	194 ± 3	80%, 606 MPa	120 ± 5
					85%, 644 MPa	123 ± 3
ESR	905 ± 7	957 ± 8	18.8 ± 1.5	171 ± 1	80%, 606 MPa	120 ± 4
					85%, 644 MPa	123 ± 3

## Data Availability

Data sharing not applicable.
